# Toxicological Effects of Roundup^®^ on *Drosophila melanogaster* Reproduction

**DOI:** 10.3390/toxics9070161

**Published:** 2021-07-07

**Authors:** Kelly Muller, Karina Herrera, Becky Talyn, Erik Melchiorre

**Affiliations:** 1 Department of Chemistry and Biochemistry, California State University, 5500 University Parkway, San Bernardino, CA 92407, USA; 004988707@coyote.csusb.edu (K.M.); 005979346@coyote.csusb.edu (K.H.); 2 Department of Biology and College of Natural Sciences, California State University, 5500 University Parkway, San Bernardino, CA 92407, USA; 3 Department of Geology, California State University, 5500 University Parkway, San Bernardino, CA 92407, USA; emelch@csusb.edu

**Keywords:** Roundup^®^, glyphosate, pelargonic acid, *Drosophila melanogaster*, reproduction

## Abstract

Herbicide use has increased dramatically since 2001, particularly Roundup^®^. Effective in agricultural practice, Roundup^®^ adversely affects non-target organisms, including reproductive and endocrine systems. We exposed fruit flies, *Drosophila melanogaster*, to either Roundup^®^ Ready to Use, containing pelargonic acid and glyphosate, or Roundup^®^ Super Concentrate, that includes glyphosate and POEA, at sublethal concentrations. Both Roundup^®^ formulations reduced ovary volume with fewer mature oocytes, most adversely at the highest concentration tested. Flies exposed within 2 h of eclosion were affected more than at 4 h, suggesting a critical period of increased ovarian sensitivity. These results support multi-species evidence that glyphosate-based herbicides interfere with normal development of the reproductive systems of non-target organisms.

## 1. Introduction

With the rise of modern agriculture, the use of genetically modified foods, pesticides, and herbicides have become ever more prevalent. Glyphosate is the active ingredient in Roundup^®^, the most commonly utilized herbicide worldwide. Glyphosate is non-selective, post-emergent, and directly obstructs plant development through the shikimate pathway which is responsible for the biosynthesis of essential aromatic amino acids in plants [1]. Much of glyphosate’s efficacy and market success can be attributed to its specificity as an inhibitor of EPSPS (enolpyruvylshikimate-3-phosphate synthase), an enzyme of the shikimate pathway [2]. As EPSPS is only found in plants and microorganisms [1], glyphosate has been branded low in toxicity to other organisms including animals. However, several studies illuminate toxic effects in a variety of species, including neurological abnormalities [3,4], oxidative stress [1,3,5], a variety of developmental defects [6,7,8,9,10,11], and more.

Roundup^®^ and other glyphosate-based herbicide (GBH) formulations have been employed at different concentrations of glyphosate, and include a variety of chemical adjuvants, secondary herbicides, and other “proprietary” ingredients. One such adjuvant is the surfactant polyethoxylated tallowamine (POEA), which increases the ability of active ingredients to penetrate leaf cuticles [12]. Although effective, commercial formulations containing surfactants may have additive or synergistic toxic effects compared to glyphosate alone [3,4,13,14,15]. Many GBHs contain secondary herbicides, such as diquat [16] or pelargonic acid, which are contact, nonselective herbicides that are only effective against actively growing, emerged vegetation [17]. Although the addition of pelargonic acid to glyphosate is not beneficial for longer-term weed control, such as regrowth control, the ready-to-use herbicides are targeted to consumers who desire earlier onset to visual plant injury [17]. While pelargonic acid toxicity is not well-studied, Techer et al. [18] reported that pelargonic acid exposure as low as 0.05 to 5 mg/L in zebrafish (*Danio rerio*) caused increased activity of glutathione peroxydase, an enzyme that protects against oxidative damage, and pelargonic acid exposure decreased circulating sex hormone levels in both male and female *D. rerio*. Additionally, pelargonic acid exposure increased mortality in the fruit fly, *Drosophila melanogaster*, both as the only active ingredient and in formulation with glyphosate [19].

Glyphosate exposure causes a variety of reproductive effects in different organisms, including changes to maternal and reproductive behavior, fecundity, and development of reproductive organs. In rodents, glyphosate altered mothers’ licking behavior towards pups [4] and increased latency time to the first mount of a mate [20]. Glyphosate decreased the fertilization of eggs laid by adult *Cantareus aspersus* exposed during embryonic development [21], and negatively impacted fecundity and fertility in the spider, *Alpaida veniliae* [7]. Glyphosate disrupts uterine development and function in rats [8,9,22], testicular development in mallard duck drakes *Anas platyrhynchos* [10], ovary and oocyte development in *A. veniliae* [7], and early development in common toads, *Bufo bufo* [6], and *D. rerio* [11].

Effects of glyphosate exposure on endocrine systems suggest that one mechanism of action causing reproductive disruption may involve interference with hormonal pathways. Glyphosate affects levels of testosterone [5,23,24,25,26], estradiol [10,20], and prolactin [5], and activates an estrogen receptor, although at relatively high concentrations compared to typical human exposure levels [27]. In contrast, rodent pups who were exposed to GBH in utero up until postnatal day 120, at 1.75 mg/kg maternal bw/day, a dose that is below the EPA limit in the U.S., showed increased anogenital distance, a marker of prenatal endocrine disruption [28]. Maternal exposure to Roundup^®^ during pregnancy and lactation delays male offspring testicular descent, which is hormonally regulated [26]. In human cell lines, glyphosate proliferated a hormone dependent human breast cancer T47D cell [15] and the authors suggest that the hormonal effects of glyphosate in postmenopausal women might induce cancer cell growth and increase breast cancer risk.

There is little conclusive evidence regarding glyphosate exposure consequences on human health due to many logistical and ethical challenges. Furthermore, it is difficult to assess individual pesticide effects in human field studies that examine general pesticide exposure, where glyphosate may be present in combination with many other agrochemicals. However, some studies show adverse hormonal and birth effects in humans. In the San Joaquin Valley of California, high pesticide exposure (95th percentile and above) increased the probability of human birth abnormality by ~9% [29]. Winchester et al. [30] observed higher rates of preterm births and shortened gestation (less than 37 weeks) with increasing pesticide use in the maternal county of residence. Early developmental exposure to pesticides increased the risk of autism spectrum disorder in children whose mothers lived within one mile of an agricultural pesticide application [31], and in children whose mothers were exposed to ambient pesticides, including glyphosate, within 2000 m of their residence [32]. Other consequences of glyphosate exposure include mutagenic and genotoxic effects on healthy human lymphocytes, oxidative stress and apoptosis on human embryonic and umbilical cells, and greater risks for premature mortality due to Parkinson’s disease [33].

*D. melanogaster* have been widely studied and used as a model organism in biomedical research for over a century [34]. *Drosophila*, which have a similar diet to humans in that they require a variety of proteins, lipids, and fats, are an advantageous model system because they are easy and inexpensive to maintain in the lab and yield large sample sizes due to their short adult lifespan and high reproductive rate [19]. Furthermore, *Drosophila* are useful in the study of human diseases as many disease genes are highly conserved between *Drosophila* and humans. In a cross-genomic analysis of 1682 human disease genes, Bier and Bodmer [35] report that 74% have homologs in *Drosophila*, with those genes causing a variety of human diseases including developmental defects, metabolic disorders, and more. A third of those genes were as highly conserved between humans and flies as functionally equivalent genes. *Drosophila* have been used as a model for human conditions such as neurodegenerative disease [36], cardiac disease [34,36], high-fat-diet-induced obesity [37], and autism [38]. Studies of glyphosate exposure in *Drosophila* show a variety of adverse effects. In *Drosophila*, glyphosate led to a reduction in ROS (reactive oxygen species) levels and an increase in the gene expression of the antioxidant defense system [1,39]. Glyphosate decreases body size and increases mortality in *Drosophila* [1,39] at concentrations as low as 1 g/L, which is within the environmentally relevant range [19]. As with other species, glyphosate showed reproductive effects in *Drosophila*, specifically a nearly complete decrease in the presence of larvae in the vials of *Drosophila* exposed to glyphosate at concentrations above 1 g/L [19].

To further explore the toxic effects of glyphosate on organisms, and more specifically, reproductive systems, we exposed females from a Canton-S strain of *Drosophila melanogaster* to the GBH Roundup^®^, and observed its effects on their ovaries.

### Specific Hypothesis

In this paper, we will report the results of experiments that test non-exclusive hypotheses about how Roundup^®^ interferes with reproduction, and begin to address the critical period of sensitivity.

**Hypothesis** **0** **(H0).** *As shown previously, Roundup^®^ reduces body size of female D. melanogaster.*

**Hypothesis** **1** **(H1).** *Roundup^®^ interferes with female Drosophila melanogaster reproduction.*

**Hypothesis** **1-A** **(H1-A).** *Roundup^®^ interferes with reproduction by reducing the size of ovaries.*

**Hypothesis** **1-B** **(H1-B).** *Roundup^®^ interferes with reproduction by reducing the number of mature oocytes.*

**Hypothesis** **2** **(H2).** *Roundup^®^ interferes with reproduction by reducing sperm production.*

## 2. Materials and Methods

### 2.1. Stock Maintenance

Stocks of the Canton-S strain of *D. melanogaster*, provided by Dr. Erik Johnson at Wake Forest University, were maintained in the laboratory in 300 mL bottles containing approximately 50 mL of medium composed of ingredients grown without pesticides, including organic corn (Bob’s Red Mill Natural Foods, Milwaukie, OR, USA), wild-collected agar (Frontier Co-op, Norway, IA, USA), non-GMO nutritional yeast (Frontier Co-op, Norway, IA, USA), and organic molasses (Wholesome Sweeteners, Sugar Land, TX, USA). Bottles were maintained at 25 °C on a 12:12 h light:dark cycle. Flies were transferred to new bottles without anesthesia, and old bottles discarded after 1 month.

Flies collected for experiments were transferred to an empty bottle and anesthetized with CO_2_, then transferred to a blue-ice pack or flypad with a continuous diffusion of CO_2_ (Genesee Scientific Corporation, El Cajon, CA, USA) to keep them asleep during sexing under a dissecting microscope.

### 2.2. Chemicals

Roundup^®^ Super Concentrate Grass and Weed Control (PCP Reg. No. 22759), manufactured by Monsanto Canada (Winnipeg, MB, Canada), was purchased from Lowes (Highland, CA, USA). From its material safety data sheet, it contains approximately 41% (41 g/100 g formulation) glyphosate (CAS number 38641-94-0), listed as the active ingredient, and 14.5% (14.5 g/100 g formulation) POEA, listed as a surfactant, and does not contain pelargonic acid (Table 1). Roundup^®^ Ready to Use Weed & Grass Killer III (EPA Reg. No. 71995-33), manufactured by Monsanto Company, Lawn & Garden Products (Marysville, OH, USA), was purchased from Lowes (Highland, CA, USA). From its material safety data sheet, it contains the active ingredients glyphosate (2%; 2 g/100 g formulation; CAS number 38641-94-0) and pelargonic acid (2%; 2 g/100 g formulation; CAS number 112-05-0) and does not include POEA (personal communication, Carly Stidam, The Scotts Company, Marysville, OH, USA; Table 1).

### 2.3. Reproductive Toxicity Experiments

In ecological settings, including agricultural and in public and private spaces, herbicides are employed in formulations containing other ingredients, rather than as the chemically pure active ingredient(s); the formulations are often kept confidential by the manufacturer. Therefore, we chose commercially available formulations to ensure that our results are ecologically relevant. This approach is consistent with many other studies in *Drosophila* [1,13,19,39] and other organisms [40,41,42,43,44,45]. The treatment vials contained food medium with either 0.0 g/L (organic control medium), 0.5, 1.0, or 2.0 g/L glyphosate acid equivalent. These concentrations were selected to minimize mortality based on LD_50_ as reported in Talyn et al. [19]. For treatments 1–3, 4.2, 8.3, or 16.7 mL of Roundup^®^ Ready to Use, which contains the active ingredients glyphosate and pelargonic acid, were added to 100 mL of medium to achieve the following final concentrations, respectively: 1.0, 2.0, and 4.0 g/L total (0.5, 1.0 and 2.0 g/L of each active ingredient, respectively). For treatments 4–6, 0.1, 0.2, or 0.5 mL of Roundup^®^ Super Concentrate with POEA, which contains glyphosate as the only active ingredient, were used per 100 mL of medium to achieve the following final concentrations, respectively: 0.5, 1.0, and 2.0 g/L.

Flies were collected within 2 or 4 h of eclosion and transferred in groups of twenty (10 of each sex) to multiple vials of each treatment containing standard medium as above, with or without herbicide. Regardless of collection time, flies were exposed to treatments for seven days. After the 7-day exposure period, the surviving flies were counted. Relative survival was calculated as:

(1) $$\mathrm{Survival}=\frac{\left(\#{\;\mathrm{flies}\;\mathrm{alive}\;\mathrm{at}\;T}_{7\;}\mathrm{in}\;\mathrm{the}\;\mathrm{treatment}\right)}{\left(\#{\;\mathrm{flies}\;\mathrm{introduced}\;\mathrm{to}\;\mathrm{the}\;\mathrm{treatment}\;\mathrm{at}\;T}_{0}\right)},$$

where T_0_ is the day the flies were transferred to the herbicide treatments and T_7_ is an observation made seven days after the flies were transferred to treatments [19]. 

After survival was scored, the males were discarded and each female was weighed intact prior to dissection. When flies were collected within 4 h of eclosion, 7–9 female flies were dissected for each treatment, including the control. For flies collected within 2 h of eclosion, we dissected a total of 18 flies for the control and dissected the following number of flies for herbicide treatments: 12, 20, and 20 for Roundup^®^ Super Concentrate with POEA at 0.5, 1.0. and 2.0 g/L glyphosate, respectively; and 10, 21, and 24 flies respectively for Roundup^®^ Ready to Use at 1.0, 2.0, and 4.0 g/L glyphosate plus pelargonic acid, respectively. Ovaries, oocytes, and spermatheca were dissected with micro forceps in Ringer’s solution under a dissecting microscope. The length and width of each ovary was measured using a stage micrometer, or using DinoCapture 2.0 Imaging Software, and the total number of mature oocytes in both ovaries or in the oviduct or uterus were counted. The volume V of each ovary was calculated using the formula for the volume of a prolate spheroid, as:

V = πLW^2^/6, (2)

where L = length and W = width of the ovary [46]. The volume of the two ovaries were averaged (in the rare cases where only one could be measured, that value was used).

During mating in *Drosophila*, sperm is transferred from the uterus to the spermathecal reservoir where it is stored for up to two weeks, coiling around the reservoir’s center and forming a toroidal mass which can be easily detected under a light microscope [47]. Thus, we observed spermatheca at 400× total magnification to visually determine the presence of sperm, indicating successful mating.

### 2.4. Statistical Analyses

All statistical analyses utilized JMP software. We first tested the data for normality using the Shapiro–Wilk test of goodness of fit to a normal distribution, as well as skewness and kurtosis, for each dependent variable (body weight, ovary volume and number of oocytes) based on the whole data set and separately for unexposed controls, Roundup^®^ Super Concentrate exposure, and Roundup^®^ Ready to Use exposure (Supplemental Table S1). Histograms of actual data with normal distributions superimposed are shown in Supplemental Figure S1. While the overall data set is non-normal (significant W), this is largely due to very low values for ovary volumes and 0 values for # oocytes in the formulation treatments, particularly Roundup^®^ Ready to Use, with correspondingly high values for both skewness and kurtosis. This type of distribution is nearly impossible to transform to a normal distribution. However, all three variables are normally distributed, or very nearly so, for unexposed controls alone. We therefore used parametric statistical tests throughout.

Survival was analyzed by Analysis of Variance (ANOVA) using three different models, one that considered the independent variables formulation and glyphosate concentration, one that considered formulation and total herbicide concentration (these two analyses required excluding the unexposed control because it necessarily occurred at only one concentration), and one that considered glyphosate concentration and pelargonic acid concentration using the number of flies surviving per vial as the unit of measure. While ANOVA takes multiple independent variables into account, since we used three different models, we applied a Bonferoni-corrected critical *p*-value for multiple analyses of the same data set of *p*_(*critical*)_ = 0.017.

For body size, ovary volume and # oocytes, individual flies are the unit of measure. We first conducted MANOVA using all variables based on individuals and the three models described for survival with exposure age added. This was repeated for each exposure age separately, and then separately for body size and for reproductive measures for flies exposed at 2 h only.

The reproductive response variables, ovary volume and number of oocytes, were analyzed first by examining their relationship to body size by calculating univariate regressions of each response variable on body size for each of the 14 treatments (*p*_(*critical*)_ = 0.0036). We then applied Multivariate ANOVA (MANOVA) using the same three models described for survival (*p*_(*critical*)_ = 0.017), followed by univariate analysis for formulation and regression analyses for continuous variables glyphosate concentration, pelargonic acid concentration, and total herbicide concentration, to address the specific hypotheses. In addition, we used one-tailed *t*-tests to determine whether each treatment X response variable combination exhibited toxic effects compared to the unexposed, organic control. Since 24 *t*-tests were performed, *p_(critical_*_)_ = 0.0021.

Finally, we converted all response variables to percent of control and repeated the analyses. This allowed us to calculate EC_50_ or the concentration of herbicide that caused a 50% reduction in body size, ovary size or number of oocytes using the equation of the regression line.

## 3. Results

**Figure 1:**
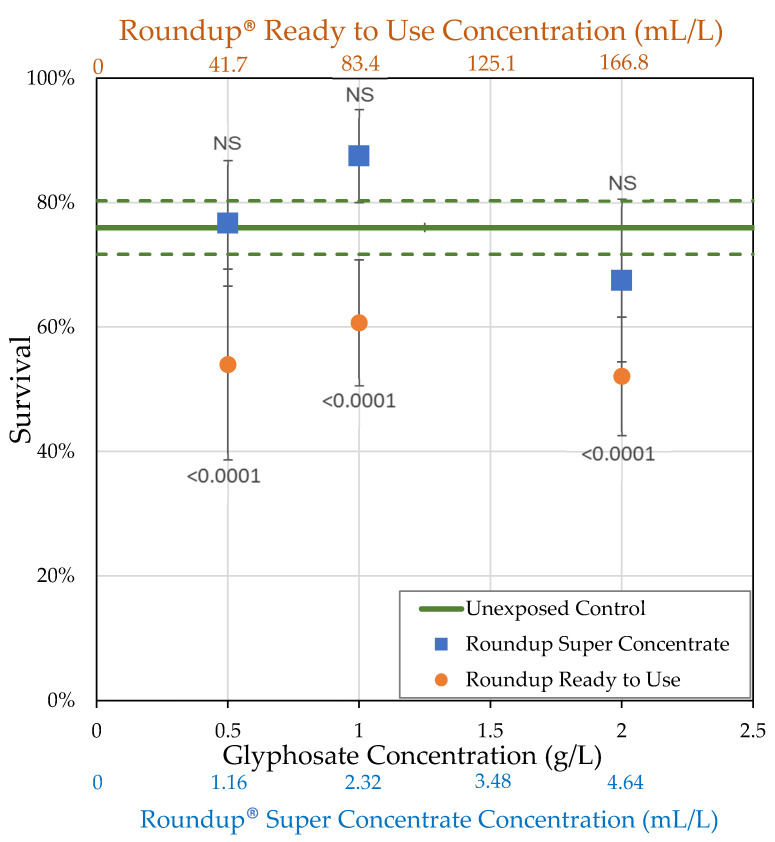
Average percent survival of *D. melanogaster* females unexposed (control) or exposed to Roundup^®^ in their food medium within 2 h of eclosion for 7 days (± std. error). The *p*-values shown are based on the *t*-tests in Table 2.

We utilized two formulations of Roundup^®^ at multiple concentrations to assess the impact of glyphosate with POEA and glyphosate + pelargonic acid formulations. After 7 days, 76% of control flies, unexposed to Roundup^®^, survived. When collected within 2 h of eclosion and observed after 7 days, survival of *Drosophila melanogaster* males and females together was not affected by Roundup^®^ Super Concentrate (*t*-tests; Figure 1; Table 2). Roundup^®^ Ready to Use had a greater effect on survival than Roundup^®^ Super Concentrate, but only increased mortality slightly compared to the organic control medium. Differences in relative survival were not statistically significant using any of the ANOVA models (Supplemental Table S2), as expected since we chose concentrations of active ingredients that were sub-lethal in a previous study [19]. Roundup^®^ exposure caused sublethal toxic effects on *D. melanogaster* females, affecting body size, ovary volume, and number of oocytes (raw data and *t*-tests, Table 2; MANOVA, Supplemental Table S3).

Exposure age slightly affected ovary volume in the models that included formulation (Supplemental Figure S2; Table S3), and formulation, glyphosate concentration and pelargonic acid concentration, but not their interactions, affected the overall models (Supplemental Table S3).

We repeated the analysis considering flies exposed at 4 h or 2 h after eclosion separately. Flies exposed to Roundup^®^ starting within 4 h of eclosion were not significantly smaller, nor were their ovary volumes or # oocytes affected by formulation, any of the herbicide concentrations, or any interactions among these variables in any of the three MANOVA models (Supplemental Table S4a). Therefore, all additional experiments were conducted using flies exposed to Roundup^®^ by 2 h after eclosion.

### 3.1. H0: As Shown Previously, Roundup^®^ Reduces Body Size of Female Drosophila Melanogaster

**Figure 2:**
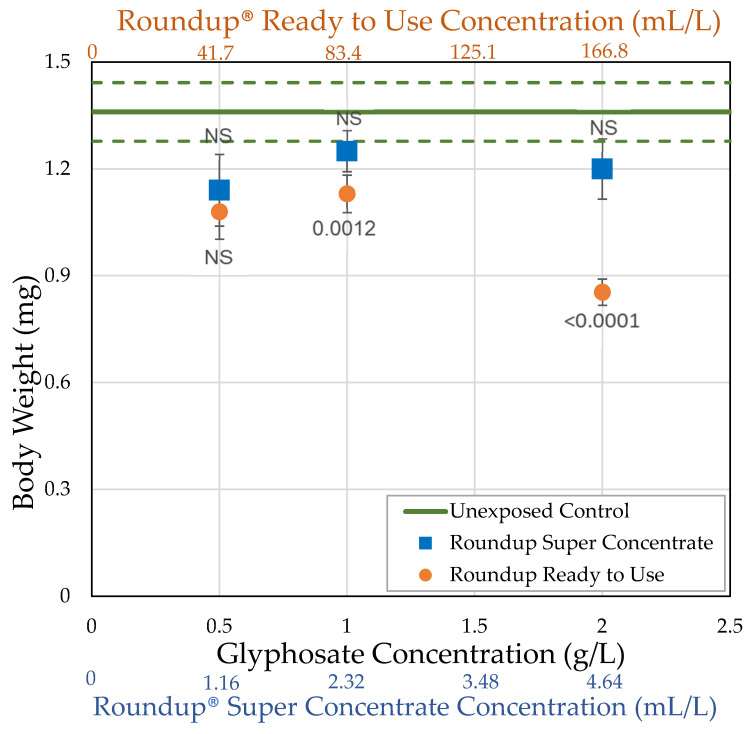
Average body weight of *D. melanogaster* females unexposed (control) or exposed to herbicide in their food medium within 2 h of eclosion for 7 days (± std. error). The *p*-values shown are based on the *t*-tests in Table 2.

Unexposed females weighed an average of 1.36 mg. When exposed within 2 h of eclosion, both Roundup^®^ formulations only minimally decreased body weight in females compared to the organic control at the lower concentrations (Figure 2; Table 2). The greatest decrease in body weight was caused by Roundup^®^ Ready to Use at 2 g/L of glyphosate or 4 g/L of combined active ingredients (glyphosate + pelargonic acid). Roundup^®^ Super Concentrate only slightly decreased body weight at the highest concentration tested, 2 g/L of active ingredient (glyphosate). Body weight depended on formulation in one ANOVA model, was weakly affected by glyphosate or pelargonic acid concentration in two models (Supplemental Table S5), and correlated with pelargonic acid, total herbicide, and Roundup^®^ Ready to Use concentration (Table 3).

### 3.2. H1: Roundup^®^ Interferes with Female Drosophila Melanogaster Reproduction

When considered together, the reproductive effects of ovary size and number of mature oocytes within the ovary differ between the two formulations, and by concentration of Roundup^®^ Ready to Use but not Roundup^®^ Super Concentrate, but no interaction between formulation and glyphosate concentration was observed. Within the same analysis, ovary volume and number of oocytes each followed the same pattern except that formulation and glyphosate concentration interacted to affect number of oocytes (MANOVA, Supplemental Table S6). Since ovary size and number of oocytes are strongly correlated with body size, we re-analyzed the results with one MANOVA to account for all three variables together (Supplemental Table S4b), and it did not substantively change the outcome.

#### 3.2.1. H1-A: Roundup^®^ Reduces Ovary Size in *Drosophila Melanogaster*

**Figure 3:**
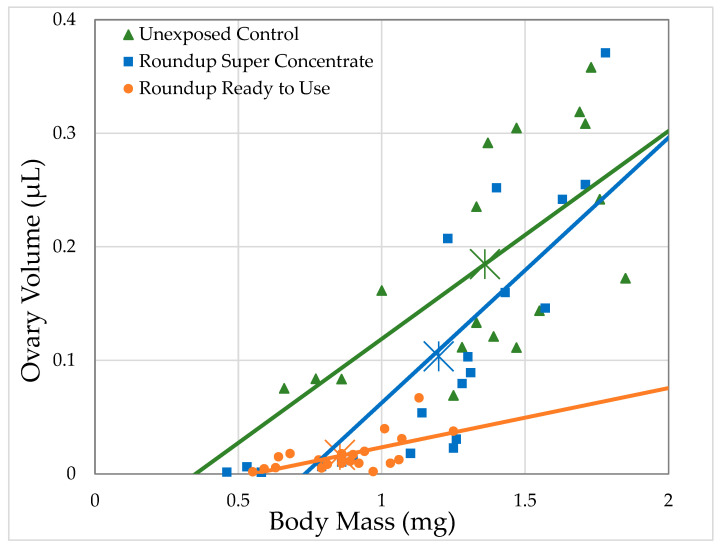
Relationship between ovary volume and body mass of *D. melanogaster* females exposed to herbicide in their food medium at the highest concentrations used (4.64 mL/L Roundup^®^ Super Concentrate and 168.8 mL/L Roundup^®^ Ready to Use; each corresponds to 2 g/L glyphosate) within 2 h of eclosion for 7 days, compared to the ovary volume of females exposed to the control medium.

Unexposed females had an average ovary volume of 184.8 nL. Ovary volume strongly correlated with body mass for unexposed controls and for both formulations at all concentrations, except Roundup^®^ Ready to Use at 0.05 g/L glyphosate, with body size explaining 29.4–66.5% of the variation in ovary volume (Generalized Regression by formulation and glyphosate concentration, Table 4). As can be seen in Figure 3, for a given body size, ovary volumes are smaller for flies exposed to Roundup^®^ Super Concentrate and, especially, for Roundup^®^ Ready to Use. While our experiment does not have the power to test this directly, it appears that for females exposed to Roundup^®^ Super Concentrate, the reduction in ovary size may account entirely or almost entirely for their smaller body size, since the largest females had both body size and ovary size well within the range of control females, and the regression lines for these two treatments converge at the larger end of the range. However, the slope of the regression line for females exposed to Roundup^®^ Ready to Use differs from the other two, reflecting that the largest female exposed at 2 g/L glyphosate (1.25 mg) was smaller than the average of unexposed females (1.36 mg), while the largest ovaries (0.067 mm^3^) from a female exposed to Roundup^®^ Ready to Use was just over 1/3 the average ovary size for unexposed females (0.19 mm^3^).

**Figure 4:**
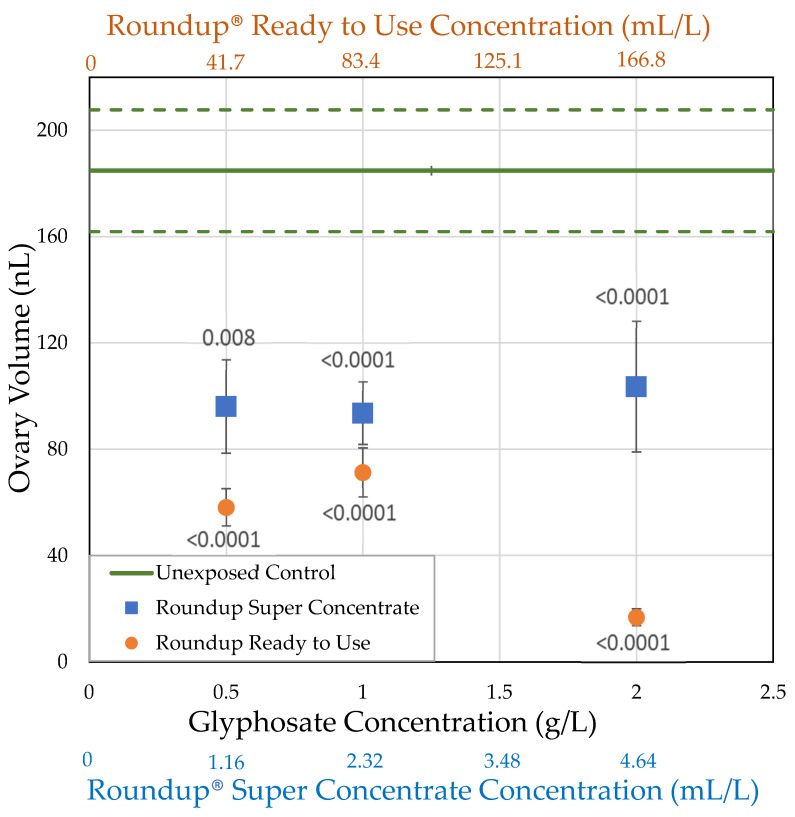
Ovary volume of *D. melanogaster* females exposed to herbicide in their food medium within 2 h of eclosion for 7 days, compared to the ovary volume of females exposed to the control medium (± std. error). The *p*-values shown are based on the *t*-tests in Table 2.

**Figure 5:**
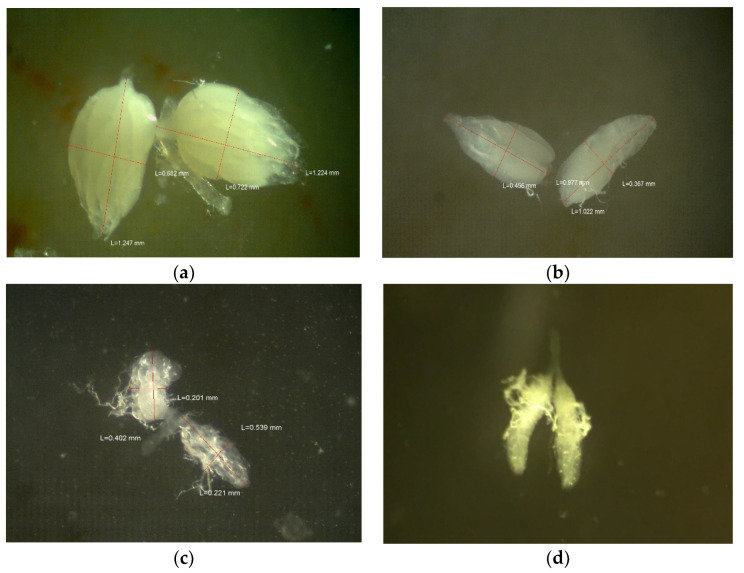
Ovary dissections of females exposed within 2 h of eclosion for 7 days to (**a**) organic control medium, (**b**) Roundup^®^ Super Concentrate at 2 g/L of glyphosate, (**c**) Roundup^®^ Ready to Use at 2 g/L of glyphosate, and (**d**) Roundup^®^ Super Concentrate at 10 g/L of glyphosate.

Both Roundup^®^ formulations, at all concentrations tested, reduced ovary size in female *Drosophila*, exposed within 2 h of eclosion for 7 days, relative to unexposed control females (*t*-tests; Figure 4 and Figure 5; Table 2). While glyphosate and Roundup^®^ Super Concentrate reduced ovary size in a non-dose-dependent manner, pelargonic acid and total herbicide concentration, as well as concentration of Roundup^®^ Ready to Use, did correlate with ovary size. When exposed to Roundup^®^ Ready to Use at 4 g/L of combined active ingredients, average ovary volume decreased to 9% of the unexposed control. Only 0.16 g/L of combined active ingredients were required, as part of the Roundup^®^ Ready to Use formulation, to reduce ovary volume to half that of controls (EC_50_, Table 3).

#### 3.2.2. H1-B: Roundup^®^ Interferes with Reproduction by Reducing the Number of Mature Oocytes

**Figure 6:**
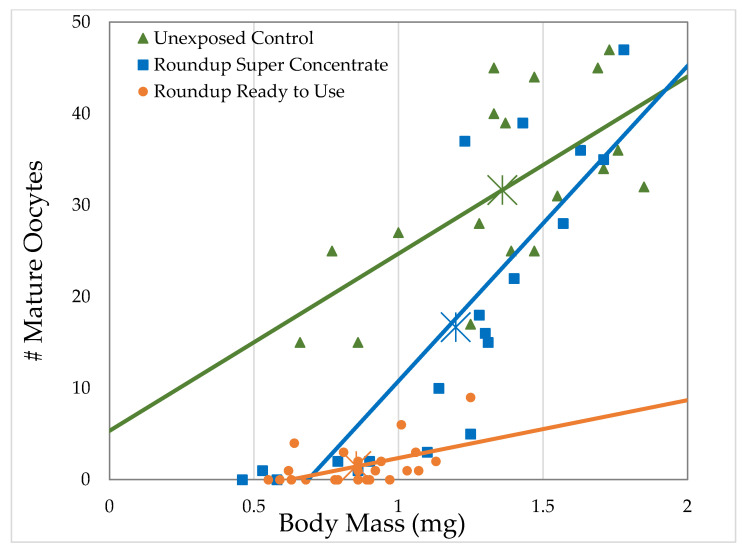
Relationship between the number of mature oocytes and body mass of *D. melanogaster* females exposed to herbicide at the highest concentrations used (4.64 mL/L Roundup^®^ Super Concentrate and 168.8 mL/L Roundup^®^ Ready to Use; each corresponds to 2 g/L glyphosate) within 2 h of eclosion for 7 days, compared to the ovary volume of females exposed to the control medium.

Unexposed females had an average of 31.7 oocytes. As with ovary volume, the number of mature oocytes strongly correlated with body mass for unexposed controls and for both formulations at all concentrations, except Roundup^®^ Ready to Use at 0.05 g/L glyphosate. Body size explained 23.2–72.8% of the variation in number of oocytes (Generalized Regression by formulation and glyphosate concentration, Table 4, Figure 6). For a given body size, female flies exposed to Roundup^®^ Super Concentrate and, especially, for Roundup^®^ Ready to Use have fewer oocytes than unexposed females. The largest females exposed to Roundup^®^ Super Concentrate had both body size and number of oocytes well within the range of control females, and the regression lines for these two treatments cross at the larger end of the range. However, the slope of the regression line for females exposed to Roundup^®^ Ready to Use differs from the other two, reflecting that the largest female exposed at 2 g/L glyphosate (1.25 mg) was smaller than the average of unexposedfemales (1.36 mg), while the female exposed to Roundup^®^ Ready to Use with the largest number of mature oocytes (9 oocytes) had 28.4% of the average number of oocytes for unexposed females (31.7 oocytes).

**Figure 7:**
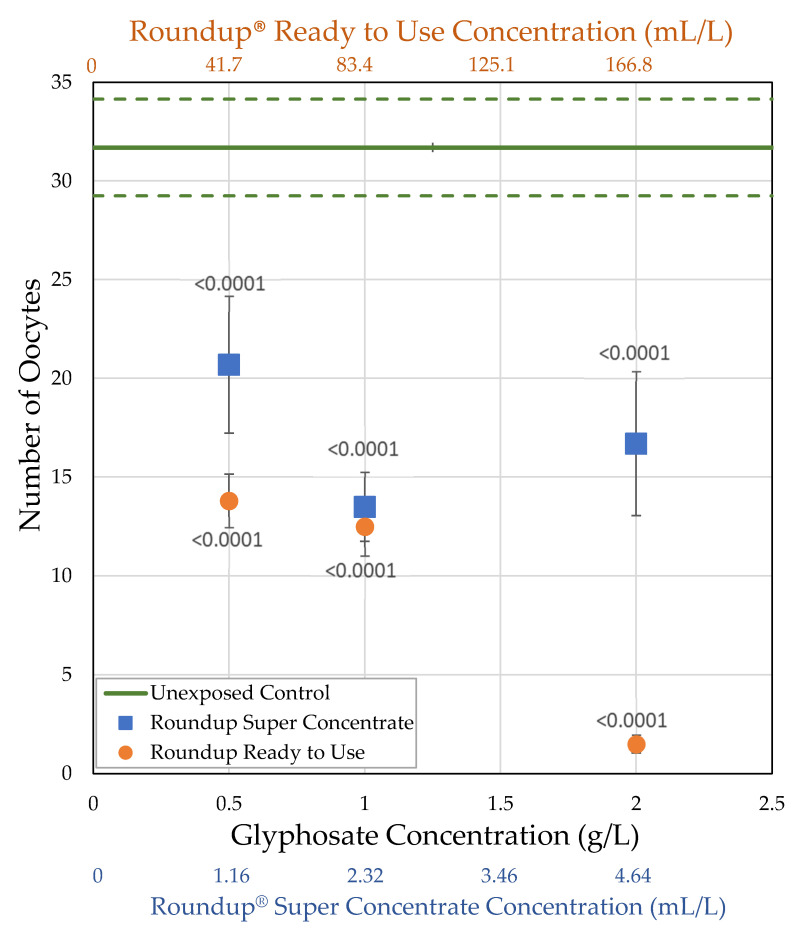
Oocytes of *D. melanogaster* females exposed to herbicide within 2 h of eclosion for 7 days, compared to the oocytes of females exposed to the control medium (± std. error).

Both Roundup^®^ formulations decreased the number of mature oocytes in female *D. melanogaster* exposed within 2 h of eclosion at all concentrations tested (*t*-tests, Table 2; Figure 5 and Figure 7). Similar to ovary size, Roundup^®^ Super Concentrate reduced the number of mature oocytes in a non-dose-dependent manner, while pelargonic acid, total herbicide, and Roundup^®^ Ready to Use concentration did correlate with the number of mature oocytes; unlike ovary size, glyphosate concentration also correlated with the number of oocytes (Linear regressions; Table 3). Only 0.96 g/L of combined active ingredients were required, as part of the Roundup^®^ Ready to Use formulation, to reduce the number of oocytes to half that of controls (EC_50_, Table 3). When exposed to Roundup^®^ Ready to use at 4 g/L of combined active ingredients, the average number of oocytes decreased to 4.7% of the unexposed control. We expected to see a correlation between ovary volume and number of oocytes, and this is the case, as females with smaller ovaries also had fewer mature oocytes (R^2^ = 65.6%, F = 270.9, *p* < 0.0001).

The combination of our results for ovary size and for number of mature oocytes indicates that Roundup^®^ has negative effects on reproductive anatomy and development in female *Drosophila*. These results support our hypothesis that Roundup^®^ reduces body size and induces reproductive toxicity in female *Drosophila* by interfering with normal ovarian development.

### 3.3. H2: Roundup^®^ Interferes with Reproduction by Reducing Sperm Production

**Figure 8:**
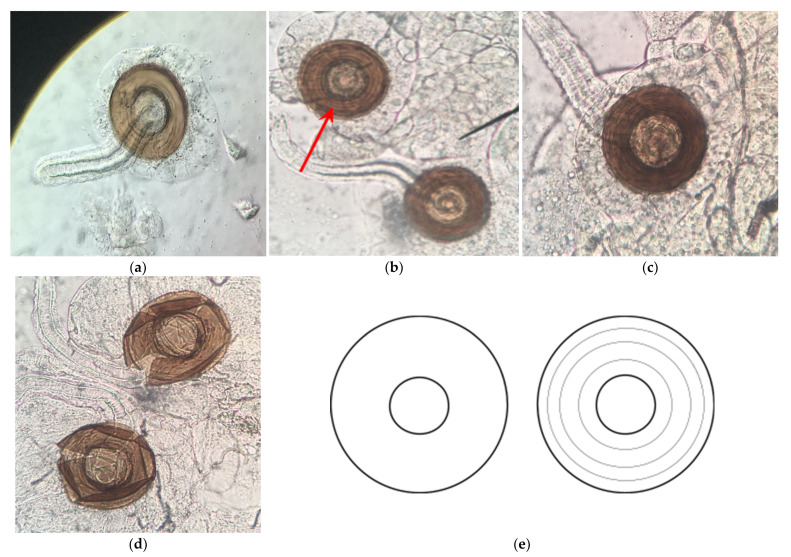
Spermatheca dissections at 400× magnification of *D. melanogaster* females exposed to (**a**) organic control medium within 4 h of eclosion, (**b**) Roundup^®^ Ready to Use at 2 g/L of glyphosate within 4 h of eclosion, (**c**) Roundup^®^ Ready to Use at 1 g/L of glyphosate within 2 h of eclosion, and (**d**) Roundup^®^ Super Concentrate at 1 g/L of glyphosate within 2 h of eclosion. The red arrow in pane (**b**) indicates one toroidal mass of sperm, which can be seen in all spermatheca we dissected. (**e**) Female *D. melanogaster* spermatheca representations: virgin (left) and mated (right).

Females of many animal species store sperm after mating [48]. In *Drosophila*, females store sperm in specialized organs, spermatheca, which can contain the sperm from multiple males and can be stored for up to two weeks [47]. The sperm is transferred from the uterus to the spermathecal reservoir where it coils around the reservoir’s center, forming a toroidal mass that can be seen under a light microscope [47]. We therefore dissected the spermatheca of female *Drosophila* from unexposed and exposed mixed-sex groups to determine the presence of sperm (Figure 8), which would indicate successful mating. We detected sperm in all spermatheca examined (Table 5), regardless of exposure to either Roundup^®^ formulation, suggesting that Roundup^®^ did not affect sperm production or interfere with mating. We cannot, however, conclude that none of the males were affected, since it may have been the case that there were enough unaffected males present to inseminate all of the females in each group. While we did not observe any empty spermatheca, we were unable to detect more subtle effects, such as lower sperm count. Therefore, more work is needed to determine whether Roundup^®^ affects male reproductive systems.

## 4. Discussion

In the reproductive toxicity experiments, survival of flies decreased when exposed to Roundup^®^ Ready to Use, which contains glyphosate and pelargonic acid but not POEA, consistent with studies showing an increase in *Drosophila* mortality after exposure to glyphosate [1,19,39]. We expected to also see a decrease in survival of flies exposed to Roundup^®^ Super Concentrate, which does contain POEA, especially since a recent study reported increased mortality in flies exposed to sub-lethal concentrations of both Roundup^®^ Concentrate Plus and POEA alone [13]. However, Roundup^®^ Super Concentrate did not affect survival at the lower concentrations and only slightly influenced survival at 2 g/L of glyphosate. These effects are small, as expected since we intentionally used concentrations below the LC_50_ for *D. melanogaster* [19].

The effects of Roundup^®^ on ovary size and number of oocytes were greater in flies exposed within 2 h of eclosion compared to 4 h of eclosion, which suggests a critical period of increased ovarian sensitivity to glyphosate. While our data do not provide evidence about the length of this period of increased sensitivity since it may begin during the larval or pupal period, the critical period ends shortly after eclosion. Further studies should examine flies exposed to GBH during pre-adult life stages, and the duration of effects after release from exposure. 

Although Roundup^®^ Super Concentrate showed minimal effects on body weight relative to flies grown on the control medium, Roundup^®^ Ready to Use did affect body weight, consistent with another study in *Drosophila* that also showed a dose dependent effect of Roundup^®^ on the weight of flies [1]. While our study did not compare the weights of males to female flies, De Aguiar et al. [1] also noted that a significant weight reduction was only observed in females, which could be explained by an effect on the gonads since the ovaries represent a significant mass compared to the total weight of the fly. Another explanation for the decrease in fly weights could be that glyphosate leads to a reduction in overall food consumption [49].

Both Roundup^®^ formulations affected ovary development at all concentrations tested, causing reduced ovary volume with fewer mature oocytes compared to the organic control. Additionally, Roundup^®^ Ready to Use reduced the number of mature oocytes in a dose-dependent manner (Table 3). Our results propose a possible explanation for those seen in two previous studies: females but not males exposed to Roundup^®^ are smaller than those that are not [1] because of a reduction in ovary size; and that the nearly complete decrease in the presence of larvae in exposed *Drosophila* [19] could result from a reduction in the number of mature oocytes after exposure to Roundup^®^. Furthermore, these results are consistent with results in other species suggesting that glyphosate and other herbicides disrupt endocrine signaling.

Roundup^®^ Ready to Use affected all parameters to a greater extent than Roundup^®^ Super Concentrate. This is consistent with previous studies showing that herbicide formulations containing pelargonic acid tend to be more toxic than those containing glyphosate as the only active ingredient [19,49]. It has been suggested that the addition of other herbicides or surfactants may increase GBH toxicity in *Drosophila* [13,19], consistent with our results that Roundup^®^ Ready to Use caused the greatest decrease in survival of flies, ovary size, and number of oocytes compared to the other herbicide treatments that contain glyphosate as the only active ingredient. Studies employing other non-target organisms, such as rodents, in addition to human cell lines, also concur [3,4,14,15]. However, further studies are needed to explore the effects of pelargonic acid and to elucidate the toxicity of POEA in combination with glyphosate.

Within agricultural sectors and in public and private spaces, herbicides and pesticides are applied in formulations containing the active ingredient(s), adjuvants/surfactants, and other ingredients often listed as proprietary information [13]. Furthermore, the composition of Roundup^®^ formulations vary depending on in which country it is sold, and often this information is not provided to the public, resulting in inconsistent data throughout the science community [50]. We chose to use formulations because these are the environmentally relevant combinations to which organisms are exposed. However, as a result of this choice, we cannot conclusively attribute our results to any one ingredient. Therefore, although the treatment containing Roundup^®^ Ready to Use did not contain POEA as a surfactant, we cannot conclude that glyphosate, or glyphosate + pelargonic acid, is solely responsible for the reproductive effects. To complicate the situation further, the formulations differ in their original concentration of active ingredients, resulting in different amounts of unknown ingredients in our final exposure treatments. Since the other ingredients in formulations are not disclosed to the public, we cannot exclude the possibility that these unknown ingredients are related to the toxic effects observed in non-target organisms, and recent studies have suggested that the formulants are more toxic than glyphosate itself [13,51]. There also may be synergistic interactions between glyphosate and these other ingredients, causing more toxic effects in combination than when used alone, and these interactions would not be accounted for by examining only single-ingredient exposures. Therefore, future studies should consider the impact of co-formulants and relevant mixtures to investigate whether glyphosate alone is responsible for any of the toxic effects reported.

## 5. Conclusions

Our results support multi-species evidence that glyphosate-based herbicides have toxic reproductive effects and interfere with normal development of the reproductive system of non-target organisms, resulting in smaller ovaries containing fewer oocytes. In addition, this study begins to establish *Drosophila melanogaster* as a model system to elucidate the mechanisms of herbicide toxicity on the reproductive system. Whether organic crops, grown without synthetic herbicides or other pesticides, are healthier than those that are conventionally grown is debated in the literature. Literature reviews [52,53,54,55] suggest that insufficient evidence is available to answer definitively, so more studies must be conducted.

## Figures and Tables

**Table 1:** Composition of the Roundup^®^ formulations used in this study.

Formulation	Active Ingredient(s) (Approximate % by Weight)		Other Ingredients (Approximate % by Weight)	
	Glyphosate	Pelargonic Acid	POEA (Surfactant)	Other Ingredients and Water
Roundup^®^ Super Concentrate Grass and Weed Control	41%	0%	14.5%	44.5%
Roundup^®^ Ready to Use Weed & Grass Killer III	2%	2%	0%	96%

**Table 2:** Average percent survival, body weight, ovary volume, and number of oocytes of *D. melanogaster* females exposed to organic control medium or herbicide treatment within 2 h of eclosion for 7 days (± std. error). Results of *t*-tests indicating which points are significantly different from the control. * *p*-values < 0.05, ** *p*-values < 0.002, the Bonferoni-corrected *p*-value for 24 *t*-tests. Active ingredients are shown in parentheses. Other known ingredients are shown in parentheses and italics.

Formulation (Active Ingredient) *(Other Known Ingredients)*	Formulation Concentration (mL/L) Glyphosate Concentration (g/L)	Survival (%)	Body Size (mg)	Ovary Volume (nL)	Number of Oocytes
Control	0.0 0.0	$\bar{x}\;$= 76.0 ± 4.30 *n* = 5	$\bar{x}$ = 1.36 ± 0.0822 *n* = 18	$\bar{x}$ = 184.8 ± 22.9 *n* = 18	$\bar{x}$ = 31.7 ± 2.45 *n* = 18
Roundup^®^ Ready to Use (glyphosate, pelargonic acid)	41.7 0.5	$\bar{x}$ = 54.0 ± 15.3 *n* = 5 t = 5.114 *p* < 0.0001 **	$\bar{x}$ = 1.08 ± 0.0771 *n* = 10 t = 2.519 *p* = 0.0059 *	$\bar{x}$ = 58.2 ± 6.98 *n* = 10 t = 4.1171 *p* < 0.0001 **	$\bar{x}$ = 13.8 ± 1.35 *n* = 10 t = 5.4456 *p* < 0.0001 **
	83.4 1.0	$\bar{x}$ = 60.7 ± 10.1 *n* = 7 t = 4.204 *p* < 0.0001 **	$\bar{x}$ = 1.13 ± 0.0524 *n* = 25 t = 3.2368 *p* = 0.0012 **	$\bar{x}$ = 71.3 ± 9.17 *n* = 21 t = 5.3487 *p* < 0.0001 **	$\bar{x}$ = 12.5 ± 1.49 *n* = 20 t = 8.28331 *p* < 0.0001 **
	166.8 2.0	$\bar{x}$ = 52.1 ± 9.50 *n* = 7 t = 6.561 *p* < 0.0001 **	$\bar{x}$ = 0.854 ± 0.0366 *n* = 25 t = 7.2448 *p* < 0.0001 **	$\bar{x}$ = 16.8 ± 3.10 *n* = 23 t = 8.2882 *p* < 0.0001 **	$\bar{x}$ = 1.50 ± 0.454 *n* = 24 t = 14.244 *p* < 0.0001 **
Roundup^®^ Super Concentrate (glyphosate) *(POEA)*	1.16 0.5	$\bar{x}$ = 76.7 ± 10.1 *n* = 3 t = 0.120 *p* = 0.548	$\bar{x}\;$= 1.14 ± 0.101 *n* = 12 t = 2.179 *p* = 0.0147 *	$\bar{x}$ = 96.1 ± 17.6 *n* = 12 t = 3.1595 *p* = 0.0008 **	$\bar{x}$ = 20.7 ± 3.47 *n* = 12 t = 3.6727 *p* < 0.0001 **
	2.32 1.0	$\bar{x}\;$= 87.5 ± 7.5 *n* = 2 t = 1.691 *p* = 0.955	$\bar{x}$ = 1.25 ± 0.0578 *n* = 21 t = 1.3878 *p* = 0.0827	$\bar{x}$ = 93.6 ± 11.8 *n* = 20 t = 4.1959 *p* < 0.0001 **	$\bar{x}$ = 13.5 ± 1.75 *n* = 20 t = 7.852 *p* < 0.0001 **
	4.64 2.0	$\bar{x}$ = 67.5 ± 13.1 *n* = 4 t = 1.767 *p* = 0.0386 *	$\bar{x}$ = 1.20 ± 0.0848 *n* = 21 t = 2.1192 *p* = 0.017 *	$\bar{x}$ = 103.6 ± 24.6 *n* = 20 t = 3.7342 *p* < 0.0001 **	$\bar{x}$ = 16.7 ± 3.65 *n* = 19 t = 6.2945 *p* < 0.0001 **

**Table 3:** Linear regression analysis of body weight, ovary volume, and number of oocytes on glyphosate, pelargonic acid, and total herbicide concentration, and for each Roundup^®^ formulation (Bivariate Fit). Regression coefficients (R^2^) and 50% Effective Concentrations (EC_50_, concentration required to induce a 50% reduction in each variable measured) are only meaningful if *p* is significant. Significant *p*-values after Bonferoni correction (*p* < 0.003) and corresponding R^2^ and EC_50_ shown in bold. * Includes unexposed controls where concentration = 0. ^1^ Sum of glyphosate concentration and pelargonic acid concentration.

Response	Independent Variable	*n*	F Ratio	*p*-Value	R^2^ (%)	EC_50_
Body weight	Glyphosate Concentration *	132	4.87	0.0294	4.17	5.31
	Pelargonic Acid Concentration *	132	23.8	**<0.0001**	**17.6**	**3.28**
	Total Herbicide Concentration *^,1^	132	19.5	**<0.0001**	**14.8**	**6.18**
	Super Concentrate [Glyphosate]	54	0.0211	0.8852	0.0405	47.01
	Ready to Use [Total Herbicide]	60	15.7	**0.0002**	**21.3**	**5.92**
Ovary Volume	Glyphosate Concentration *	124	2.77	0.099	2.6	0.12
	Pelargonic Acid Concentration *	124	27.5	**<0.0001**	**20.9**	**0.13**
	Total Herbicide Concentration *^,1^	124	18.5	**<0.0001**	**15.1**	**1.02**
	Super Concentrate [Glyphosate]	52	0.122	0.728	0.243	0.39
	Ready to Use [Total Herbicide]	54	27.1	**<0.0001**	**34.2**	**0.16**
Number of Oocytes	Glyphosate Concentration *	123	11.9	**0.0008**	**10.3**	**0.63**
	Pelargonic Acid Concentration *	123	39.6	**<0.0001**	**27.8**	**0.15**
	Total Herbicide Concentration *^,1^	123	36.6	**<0.0001**	**26.2**	**1.16**
	Super Concentrate [Glyphosate]	51	0.131	0.719	0.267	1.73
	Ready to Use [Herbicide]	54	72.7	**<0.0001**	**58.3**	**0.96**

**Table 4:** Linear regression of ovary volume and number of oocytes on body size, by formulation and glyphosate concentration. Regression coefficients (R^2^) are only meaningful if *p* < 0.05, shown in bold.

Formulation	Formulation Concentration (mL/L)/Glyphosate Concentration (g/L)	Ovary Volume				# Oocytes			
		*n*	R^2^ (%)	χ^2^	*p*-Value	*n*	R^2^ (%)	χ^2^	*p*-Value
Unexposed Control	0/0	18	**43.2**	12.2	**0.0005**	18	**42.3**	11.8	**0.0006**
Ready to Use	41.7/0.5	19	5.52	0.467	0.494	19	2.25	0.184	0.668
	83.4/1	35	**32.9**	9.33	**0.0023**	35	**35.9**	11.65	**0.0006**
	166.8/2	34	**39**	13.4	**0.0003**	34	**23.2**	7.94	**0.0048**
Super Concentrate	1.16/0.5	22	**54.6**	14.2	**0.0002**	22	**55**	12.2	**0.0005**
	2.32/1	31	**29.4**	7.48	**0.0062**	31	**25**	6	**0.0143**
	4.64/2	30	**66.5**	35.8	**<0.0001**	30	**72.8**	45.5	**<0.0001**

**Table 5:** Number of *D. melanogaster* female spermatheca observations per exposure treatment.

Formulation	Formulation Concentration (mL/L)/ Glyphosate Concentration (g/L)	Glyphosate Concentration (g/L)	*n* at 4 h	*n* at 2 h
Unexposed Control	0/0	0	3	1
Roundup^®^ Super Concentrate	41.7/0.5	0.5	3	4
	83.4/1	1.0	3	6
	166.8/2	2.0	3	0
Roundup^®^ Ready to Use	1.16/0.5	0.5	3	0
	2.32/1	1.0	3	1
	4.64/2	2.0	3	0

## Data Availability

Data are available upon request, by sending an email to the corresponding author at btalyn@csusb.edu.

## Supplementary Materials

The following are available online at https://www.mdpi.com/article/10.3390/toxics9070161/s1, Figure S1: Distribution of Dependent Variables. Bars indicate the distribution of each dependent variable: (a) survival, (b) body weight, (c) ovary volume, (d) number of oocytes. In each case, the left panel shows the distribution of all exposure treatments combined with controls indicated by diagonal lines and all others as solid, and the right panel shows the distribution of controls only with the normal distribution fitted only to those data. Lines indicate the normal distribution, and box plots of the same data are shown to the right of each histogram. Figure S2. Comparison of responses to Roundup^®^ exposure starting at 2 vs. 4 h after eclosion. Average body weight (a), ovary volume (b), and number of oocytes (c) of *D. melanogaster* females exposed to Roundup Super Concentrate (blue) or Roundup^®^ Ready to Use (orange) in their food medium within either 2 h (circle) or 4 h (square) of eclosion for 7 days (± std. error). Table S1: Test for normality (goodness of fit test, W and *p* < W), skewness, and kurtosis for all three dependent variables based on the whole data set (overall) and each of the three exposure treatments (unexposed controls, exposed to Roundup^®^ Super Concentrate, and exposed to Roundup^®^ Ready to Use). Bold indicates significant *p*-values < 0.0031. Table S2. ANOVAs for survival of flies exposed to Roundup^®^ starting by 2 h after eclosion. These ANOVAs test the effects of glyphosate concentration, pelargonic acid concentration, formulation, herbicide concentration, and the interaction between these on survival of flies exposed to herbicide within 2 h of eclosion. ANOVA uses least square means identity matrix. Model 1 includes data from controls, while models 2 and 3 necessarily exclude these because of the interaction between formulation and concentration. For whole model, F-value based on Wilks’ Lambda. Other F-values based on Exact F Test. Significant *p*-values after Bonferoni correction for three models (*p* < 0.017) shown in bold. Table S3. MANOVAs for all Roundup^®^ exposure and age treatments combined. Results of MANOVAs testing the effects of exposure age, formulation, total herbicide concentration, glyphosate concentration, pelargonic acid concentration and the interaction between these on body weight, ovary volume and number of mature oocytes. MANOVA uses least square means identity matrix. Model 1 includes data from controls, while models 2 and 3 necessarily exclude these because of the interaction between formulation and concentration. For whole model, F-value based on Wilks’ Lambda. Other F-values based on Exact F Test. Significant *p*-values after Bonferoni correction for three models (*p* < 0.017) shown in bold. Table S4. Separate MANOVAs for exposure to Roundup^®^ starting at 4 h or 2 h after eclosion. Results of MANOVAs testing the effects of formulation, total herbicide concentration, glyphosate concentration, pelargonic acid concentration, and the interaction between these on body weight, ovary volume, and number of oocytes of flies exposed to herbicide within 4 h of eclosion (a) or 2 h of eclosion (b). Least squares, mean, identity matrix. Model 1 includes data from controls, while models 2 and 3 necessarily exclude these because of the interaction between formulation and concentration. For whole models, F-ratio based on Wilks’ Lambda. Other F-values based on Exact F Test. Significant *p*-values after Bonferoni correction for three models (*p* < 0.017) shown in bold. Table S5. ANOVAs for body weight of flies exposed to Roundup^®^ starting by 2 h after eclosion. Results of ANOVAs testing the effects of formulation, total herbicide concentration, glyphosate concentration, pelargonic acid concentration, and the interaction between these on body weight of flies exposed to herbicide within 2 h of eclosion (DF = 127). ANOVA uses least square means identity matrix. For whole model, F-value based on Wilks’ Lambda. Other F-values based on Exact F Test. Significant *p*-values after Bonferoni correction for three models (*p* < 0.017) shown in bold. Table S6. MANOVAs for reproductive response variables of flies exposed to Roundup^®^ starting by 2 h after eclosion. Results of MANOVAs testing the effects of formulation, total herbicide concentration, glyphosate concentration, pelargonic acid concentration, and the interaction between these on the reproductive response variables (ovary size and number of oocytes) on flies exposed to herbicide within 2 h of eclosion. MANOVA uses least square means identity matrix. For whole model, F-value based on Wilks’ Lambda. Other F-values based on Exact F Test. Significant *p*-values after Bonferoni correction for two models (overall and by formulation; *p* < 0.025) shown in bold.
