# Supplementary material for: Toxicological Effects of Roundup® on Drosophila melanogaster Reproduction
Source: Toxics. 2021 Jul 7;9(7):161. doi: 10.3390/toxics9070161 (PMC8309847; doi:10.3390/toxics9070161)
Supplement: Supplementary file 1 [file toxics-09-00161-s001.zip › toxics-1259094-supplementary.pdf]

toxics

MDPI

Article

Supplementary Material: Toxicological Effects of Roundup® on *Drosophila melanogaster* Reproduction

Kelly Muller, Karina Herrera, Becky Talyn and Erik Melchiorre

Table S1. Test for normality (goodness of fit test, W and $p < W$), skewness, and kurtosis for all three dependent variables based on the whole data set (overall) and each of the three exposure treatments (unexposed controls, exposed to Roundup® Super Concentrate, and exposed to Roundup® Ready to Use). Bold indicates significant p-values < 0.0031.

Exposure Treatment	Test of Normality	Test Statistic	Survival (Proportion Per Vial)	Body Weight (mg)	Ovary Volume (nL)	Oocytes (Number)
Overall	goodness of fit to	W	0.874	0.563	0.355	0.392
	normal distribution	*p*	0.0012	<0.0001	<0.0001	<0.0001
	Skewness		−0.807	−0.181	1.65	1.2
	Kurtosis		−0.626	−0.509	2.97	1.73
Controls	goodness of fit to	W	0.859	0.935	0.893	0.942
	normal distribution	*p*	0.223	0.238	0.0426	0.31
	skewness		−1.518	−0.621	0.475	−0.128
	Kurtosis		2.608	−0.381	−1.33	−1.03
Roundup® Super Concentrate	goodness of fit to	W	0.856	0.972	0.898	0.948
	normal distribution	*p*	0.0856	0.0675	<0.0001	0.0041
	skewness		−0.605	−0.402	1.189	0.769
	Kurtosis		−1.270	−0.398	1.25	0.337
Roundup® Ready to Use	goodness of fit to	W	0.834	0.98	0.804	0.808
	normal distribution	*p*	0.0038	0.206	<0.0001	<0.0001
	skewness		−0.466	−0.211	2.07	2.12
	Kurtosis		−1.598	−0.707	5.87	7.92

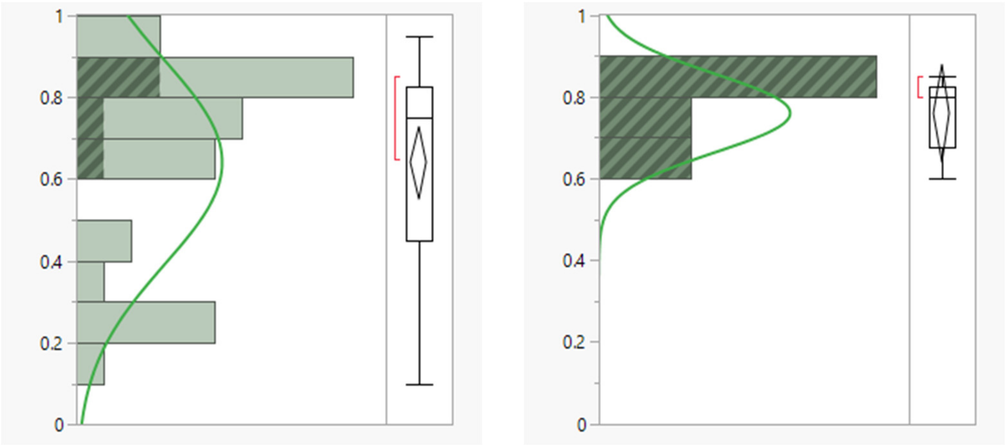

(**a**) Survival

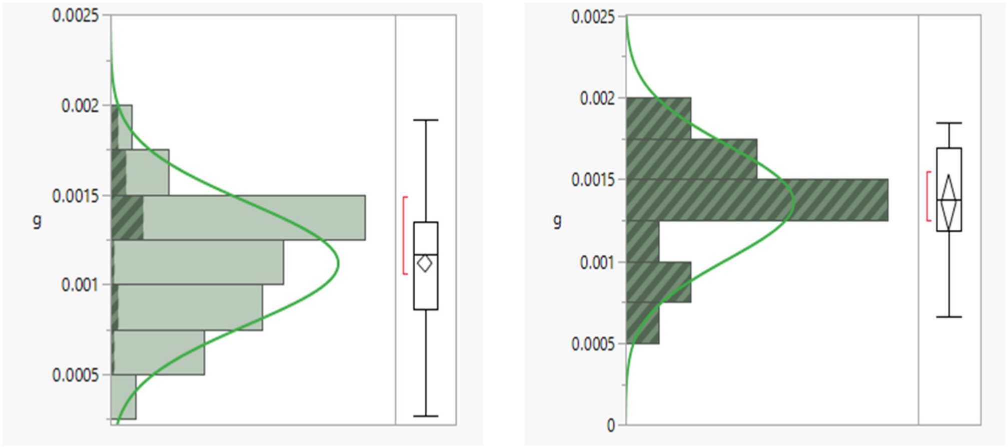

(**b**) Body Weight

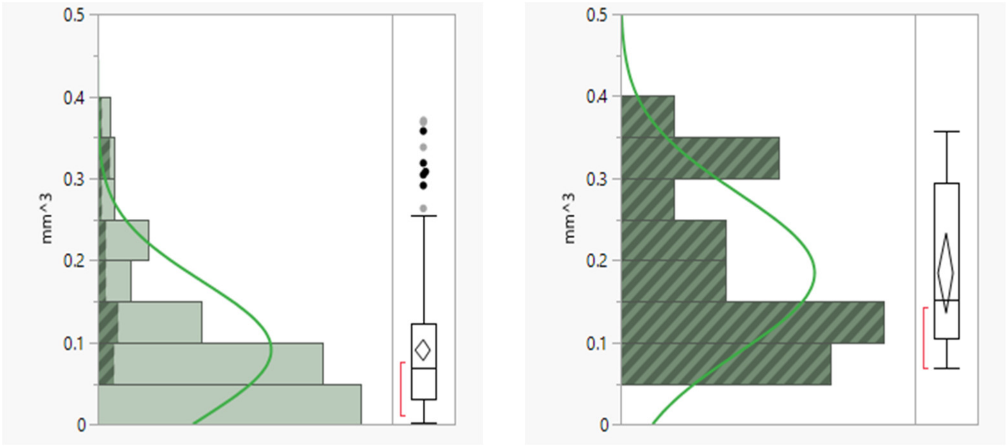

(**c**) Ovary Volume

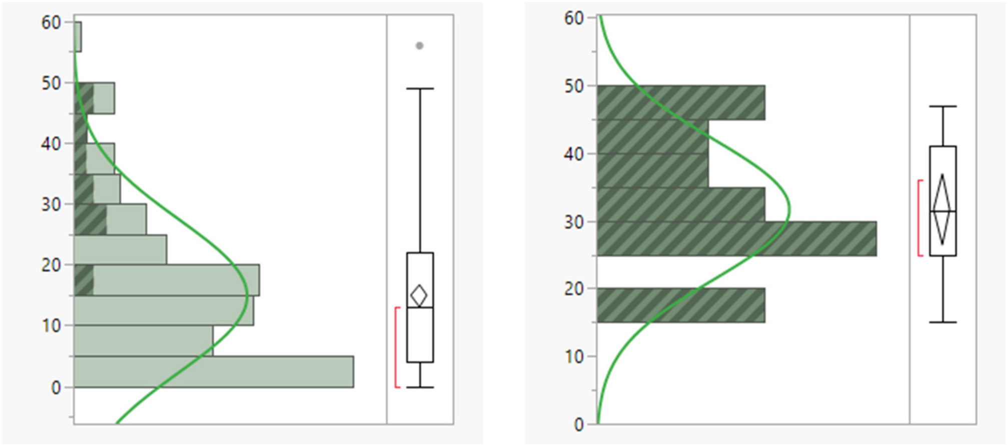

(**d**) Number of Oocytes

Figure S1. Distribution of Dependent Variables. Bars indicate the distribution of each dependent variable: (**a**) survival, (**b**) body weight, (**c**) ovary volume, (**d**) number of oocytes. In each case, the left panel shows the distribution of all exposure treatments combined with controls indicated by diagonal lines and all others as solid, and the right panel shows the distribution of controls only with the normal distribution fitted only to those data. Lines indicate the normal distribution, and box plots of the same data are shown to the right of each histogram.

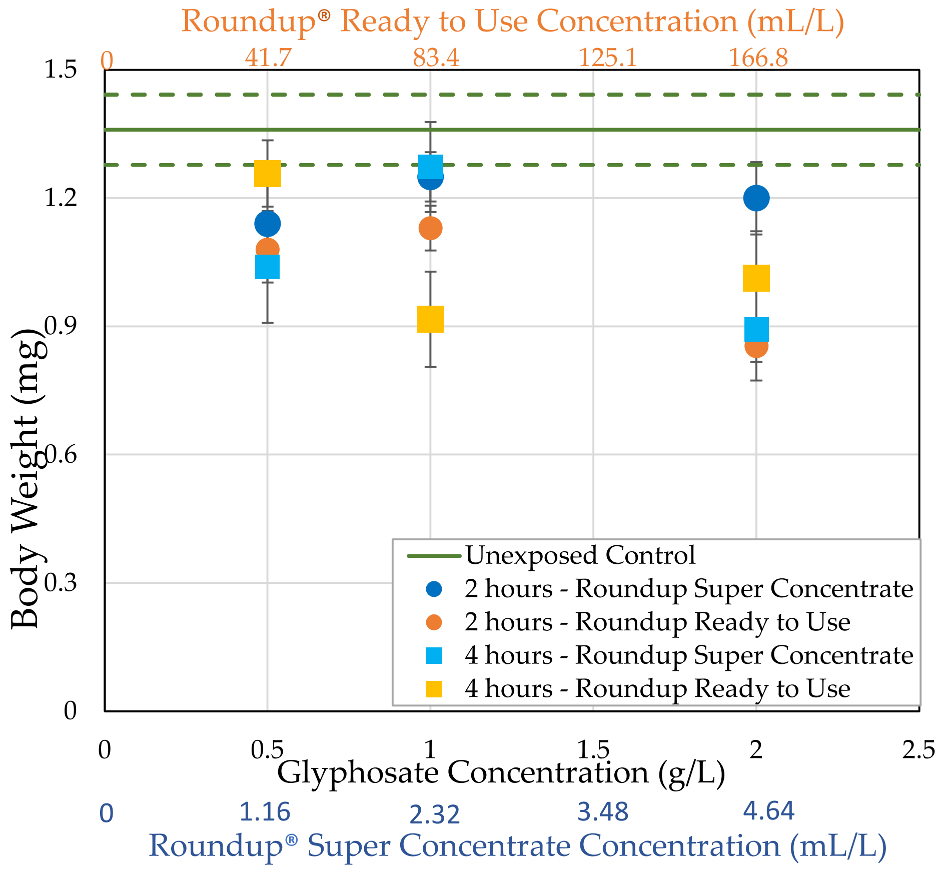

Roundup® Ready to Use Concentration (mL/L)
0
41.7
83.4
125.1
166.8
1.5
1.2
0.9
0.6
0.3
0
Body Weight (mg)
Unexposed Control
2 hours - Roundup Super Concentrate
2 hours - Roundup Ready to Use
4 hours - Roundup Super Concentrate
4 hours - Roundup Ready to Use
0
0.5
1
1.5
2
2.5
Glyphosate Concentration (g/L)
0
1.16
2.32
3.48
4.64
Roundup® Super Concentrate Concentration (mL/L)

(**a**)

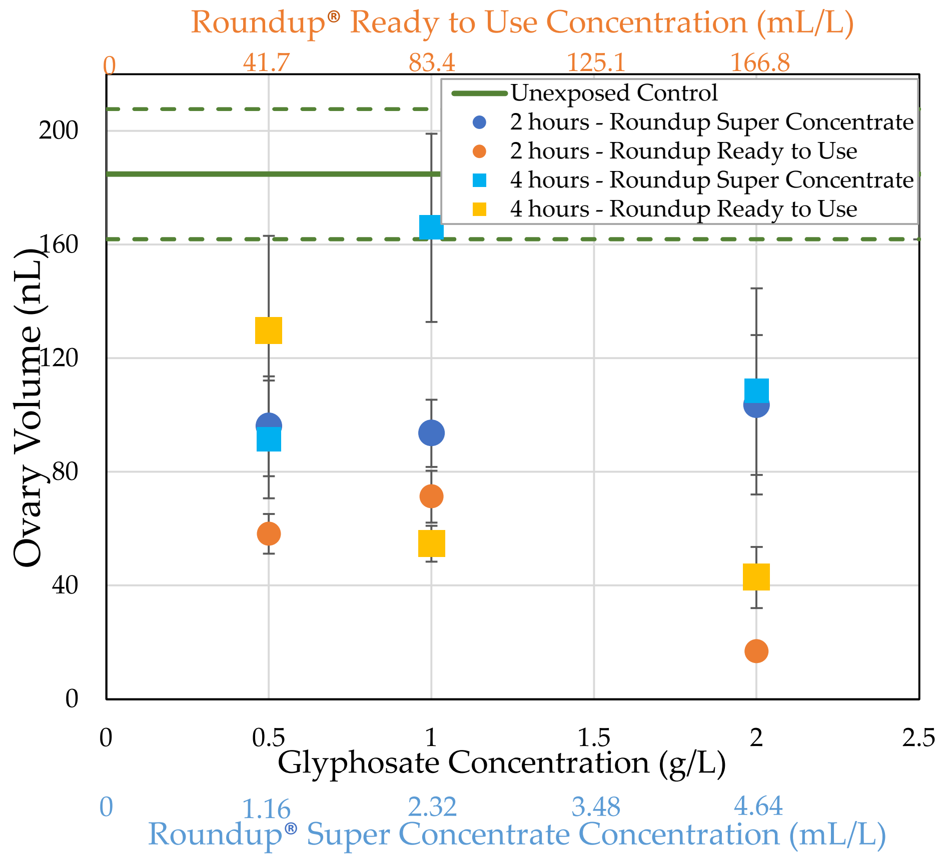

Roundup® Ready to Use Concentration (mL/L)
0
41.7
83.4
125.1
166.8
200
160
120
80
40
0
Ovary Volume (nL)
Unexposed Control
2 hours - Roundup Super Concentrate
2 hours - Roundup Ready to Use
4 hours - Roundup Super Concentrate
4 hours - Roundup Ready to Use
0
0.5
1
1.5
2
2.5
Glyphosate Concentration (g/L)
0
1.16
2.32
3.48
4.64
Roundup® Super Concentrate Concentration (mL/L)

(**b**)

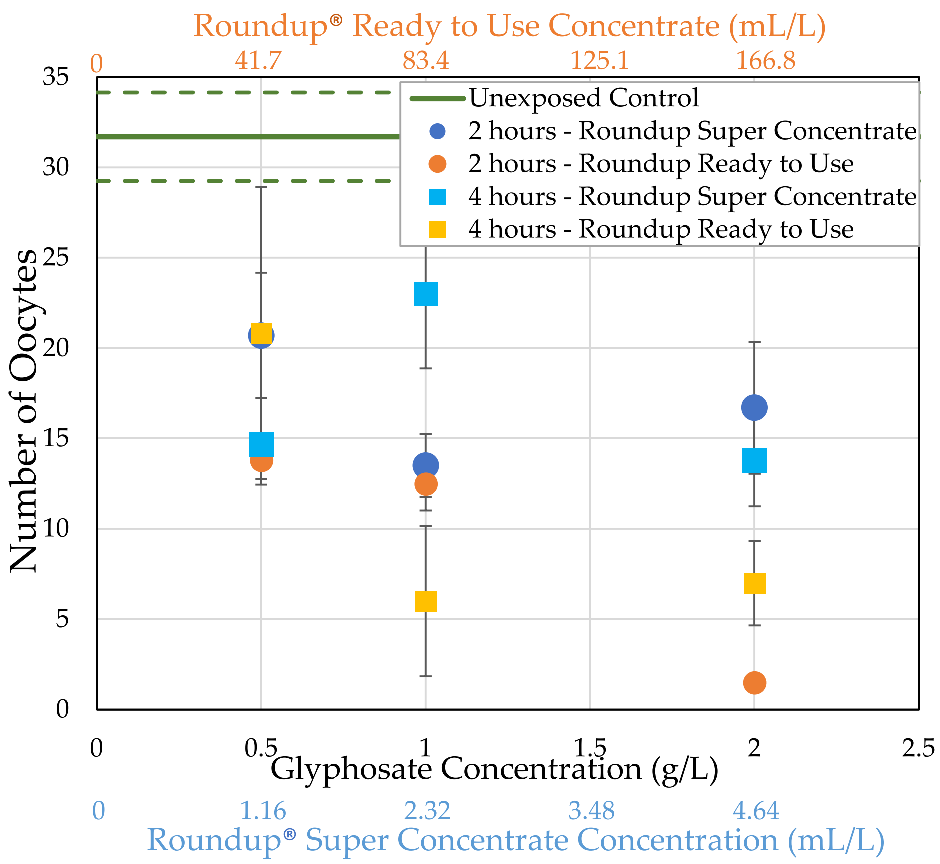

(**c**)

Figure S2. Comparison of responses to Roundup® exposure starting at 2 vs. 4 hours after eclosion. Average body weight (**a**), ovary volume (**b**), and number of oocytes (**c**) of *D. melanogaster* females exposed to Roundup Super Concentrate (blue) or Roundup® Ready to Use (orange) in their food medium within either 2 hours (circle) or 4 hours (square) of eclosion for 7 days (± std. error).

Table S2. ANOVAs for survival of flies exposed to Roundup® starting by 2 hours after eclosion. These ANOVAs test the effects of glyphosate concentration, pelargonic acid concentration, formulation, herbicide concentration, and the interaction between these on survival of flies exposed to herbicide within 2 hours of eclosion. ANOVA uses least square means identity matrix. Model 1 includes data from controls, while models 2 and 3 necessarily exclude these because of the interaction between formulation and concentration. For whole model, F-value based on Wilks' Lambda. Other F-values based on Exact F Test. Significant *p*-values after Bonferoni correction for three models ($p < 0.017$) shown in bold.

**Model (DF)**	**Independent Variable**	**F**	***p***
1 (29)	Whole model	1.60	0.212
	Glyphosate Concentration	0.22	0.641
	Pelargonic Acid Concentration	2.96	0.096
	Glyphosate Concentration X Pelargonic Acid Concentration	0.72	0.404
2 (24)	Whole model	1.28	0.304
	Formulation	0.66	0.423
	Herbicide Concentration	0.48	0.497
	Formulation X Herbicide Concentration	0.23	0.638
3 (24)	Whole model	1.28	0.304
	Formulation	3.45	0.076
	Glyphosate Concentration	0.46	0.503
	Glyphosate Concentration X Pelargonic Acid Concentration	0.10	0.755

Table S3. MANOVAs for all Roundup® exposure and age treatments combined. Results of MANOVAs testing the effects of exposure age, formulation, total herbicide concentration, glyphosate concentration, pelargonic acid concentration and the interaction between these on body weight, ovary volume and number of mature oocytes. MANOVA uses least square means identity matrix. Model 1 includes data from controls, while models 2 and 3 necessarily exclude these because of the interaction between formulation and concentration. For whole model, F-value based on Wilks' Lambda. Other F-values based on Exact F Test. Significant *p*-values after Bonferoni correction for three models ($p < 0.017$) shown in bold.

Response Variable	Model (DF)	Independent Variable	F	*p*
Overall	1 (139)	Whole Model	4.86	**<0.0001**
		Exposure Age	2.02	0.113
		Formulation	4.08	**0.0083**
		Herbicide Concentration	4.94	**0.0027**
		Formulation X Herbicide Concentration	1.61	0.189
	2 (139)	Whole Model	4.86	**<0.0001**
		Exposure Age	2.02	0.113
		Formulation	7.37	**0.0001**
		Glyphosate Concentration	6.92	**0.0002**
		Formulation X Glyphosate Concentration	2.78	0.0433
	3 (157)	Whole Model	6.83	**<0.0001**
		Exposure Age	0.823	0.483
		Glyphosate Concentration	4.84	**0.003**
		Pelargonic Acid Concentration	6.65	**0.0003**
		Glyphosate Concentration X Pelargonic Acid Concentration	1.37	0.254
Body Weight	1 (139)	Whole Model	3.78	**0.006**
		Exposure Age	0.0055	0.941
		Formulation	0.0094	0.923
		Herbicide Concentration	5.49	0.0205
		Formulation X Herbicide Concentration	0.25	0.618
	2 (139)	Whole Model	3.78	**0.006**
		Exposure Age	0.0055	0.941
		Formulation	3.21	0.075
		Glyphosate Concentration	8.82	**0.0035**
		Formulation X Glyphosate Concentration	2.3	0.132
	3 (157)	Whole Model	5.9	**0.0002**
		Exposure Age	0.0125	0.911
		Glyphosate Concentration	5.82	**0.017**
		Pelargonic Acid Concentration	1.26	0.264
		Glyphosate Concentration X Pelargonic Acid Concentration	0.104	0.747
Ovary Volume	1 (139)	Whole Model	9.36	**<0.0001**
		Exposure Age	4.06	0.046
		Formulation	7.21	**0.0081**
		Herbicide Concentration	0.878	0.35
		Formulation X Herbicide Concentration	4.04	0.0463
	2 (139)	Whole Model	9.36	**<0.0001**
		Exposure Age	4.06	0.0457
		Formulation	18.9	**<0.0001**
		Glyphosate Concentration	3.62	0.0593
		Formulation X Glyphosate Concentration	7.56	**0.0068**
	3 (157)	Whole Model	12.1	**<0.0001**
		Exposure Age	1.31	0.254

		Glyphosate Concentration	1.25	0.266
		Pelargonic Acid Concentration	14.4	**0.0002**
		Glyphosate Concentration X Pelargonic Acid Concentration	1.49	0.224
Number of Oocytes	1 (139)	Whole Model	11.51	**<0.0001**
		Exposure Age	1.87	0.1738
		Formulation	2.01	0.159
		Herbicide Concentration	8.14	**0.005**
		Formulation X Herbicide Concentration	1.78	0.185
	2 (139)	Whole Model	11.5	**<0.0001**
		Exposure Age	1.87	0.174
		Formulation	17.8	**<0.0001**
		Glyphosate Concentration	15.2	**0.0001**
		Formulation X Glyphosate Concentration	7.31	**0.0077**
	3 (157)	Whole Model	19.8	**<0.0001**
		Exposure Age	0.342	0.559
		Glyphosate Concentration	8.22	**0.0047**
		Pelargonic Acid Concentration	15.7	**0.0001**
		Glyphosate Concentration X Pelargonic Acid Concentration	1.83	0.178

Table S4. Separate MANOVAs for exposure to Roundup® starting at 4 hours or 2 hours after eclosion. Results of MANOVAs testing the effects of formulation, total herbicide concentration, glyphosate concentration, pelargonic acid concentration, and the interaction between these on body weight, ovary volume, and number of oocytes of flies exposed to herbicide within 4 h of eclosion (a) or 2 h of eclosion (b). Least squares, mean, identity matrix. Model 1 includes data from controls, while models 2 and 3 necessarily exclude these because of the interaction between formulation and concentration. For whole models, F-ratio based on Wilks' Lambda. Other F-values based on Exact F Test. Significant *p*-values after Bonferoni correction for three models ($p < 0.017$) shown in bold.

(a) Flies Exposed within 4 h of Eclosion				
**Response Variable**	**Model (DF)**	**Independent Variable**	**F**	***p***
Overall	1 (34)	Whole Model	1.85	0.0706
		Formulation	3.17	0.0367
		Herbicide Concentration	1.64	0.199
		Formulation X Herbicide Concentration	0.873	0.465
	2 (34)	Whole Model	1.85	0.071
		Formulation	3.08	0.0404
		Glyphosate Concentration	1.79	0.168
		Formulation X Glyphosate Concentration	0.87	0.468
	3 (34)	Whole Model	1.9	0.063
		Glyphosate Concentration	2.38	0.0871
		Pelargonic Acid Concentration	2.03	0.128
		Glyphosate Concentration X Pelargonic Acid Concentration	0.337	0.798
Body Weight	1 (36)	Whole Model	2.02	0.129
		Formulation	5.24	0.028
		Herbicide Concentration	3.85	0.576
		Formulation X Herbicide Concentration	0.328	0.57
	2 (36)	Whole Model	2.02	0.129
		Formulation	2.08	0.158
		Glyphosate Concentration	4.24	0.0467
		Formulation X Glyphosate Concentration	0.0089	0.926
	3	Whole Model	2.05	0.124

	(36)	Glyphosate Concentration	6.02	0.0191
		Pelargonic Acid Concentration	1.82	0.185
		Glyphosate Concentration X Pelargonic Acid Concentration	0.641	0.429
Ovary Volume	1<br>(36)	Whole Model	1.9	0.148
		Formulation	0.4	0.532
		Herbicide Concentration	0.518	0.476
		Formulation X Herbicide Concentration	1	0.324
	2<br>(36)	Whole Model	1.9	0.148
		Formulation	2.09	0.157
		Glyphosate Concentration	1.5	0.229
		Formulation X Glyphosate Concentration	2.08	0.158
	3<br>(36)	Whole Model	1.18	0.169
		Glyphosate Concentration	0.111	0.741
		Pelargonic Acid Concentration	0.75	0.392
		Glyphosate Concentration X Pelargonic Acid Concentration	0.065	0.8
Number of Oocytes	1<br>(36)	Whole Model	2.04	0.125
		Formulation	0.0002	0.988
		Herbicide Concentration	2.45	0.126
		Formulation X Herbicide Concentration	0.078	0.782
	2<br>(36)	Whole Model	2.04	0.125
		Formulation	1.49	0.23
		Glyphosate Concentration	3.72	0.0616
		Formulation X Glyphosate Concentration	0.868	0.358
	3<br>(36)	Whole Model	2.03	0.127
		Glyphosate Concentration	0.849	0.363
		Pelargonic Acid Concentration	0.971	0.331
		Glyphosate Concentration X Pelargonic Acid Concentration	0.0306	0.862
		**(b) Flies Exposed within 2 h of Eclosion**		
**Response Variable**	**Model (DF)**	**Independent Variable**	**F**	***p***
Overall	1<br>(98)	Whole Model	5.47	**<0.0001**
		Formulation	3.94	**0.0106**
		Herbicide Concentration	5.31	**0.002**
		Formulation X Herbicide Concentration	1.05	0.373
	2<br>(98)	Whole Model	5.47	**<0.0001**
		Formulation	6.67	**0.0004**
		Glyphosate Concentration	7.7	**0.0001**
		Formulation X Glyphosate Concentration	2.32	0.0802
	3<br>(116)	Whole Model	8.81	**<0.0001**
		Glyphosate Concentration	5	**0.0027**
		Pelargonic Acid Concentration	5.15	**0.0022**
		Glyphosate Concentration X Pelargonic Acid Concentration	1.42	0.242
Body Weight	1<br>(100)	Whole Model	6.61	**0.0004**
		Formulation	2.23	0.139
		Herbicide Concentration	2.18	0.149
		Formulation X Herbicide Concentration	1.15	0.287
	2<br>(100)	Whole Model	6.61	**0.0004**
		Formulation	10.5	**0.0016**
		Glyphosate Concentration	4.67	0.0331

		Formulation X Glyphosate Concentration	3.45	0.0664
		Whole Model	9.32	**<0.0001**
	3 (118)	Glyphosate Concentration	1.82	0.18
		Pelargonic Acid Concentration	4.45	0.037
		Glyphosate Concentration X Pelargonic Acid Concentration	0.313	0.86
Ovary Volume	1 (100)	Whole Model	9.43	**<0.0001**
		Formulation	8.56	**0.0042**
		Herbicide Concentration	0.345	0.558
		Formulation X Herbicide Concentration	3.14	0.0795
	2 (100)	Whole Model	9.43	**<0.0001**
		Formulation	19.7	**<0.0001**
		Glyphosate Concentration	1.97	0.163
		Formulation X Glyphosate Concentration	5.5	0.021
	3 (118)	Whole Model	15.1	**<0.0001**
		Glyphosate Concentration	1.26	0.264
		Pelargonic Acid Concentration	13.9	**0.0003**
		Glyphosate Concentration X Pelargonic Acid Concentration	2.02	0.158
Number of Oocytes	1 (100)	Whole Model	13.1	**<0.0001**
		Formulation	3.04	0.0841
		Herbicide Concentration	5.3	0.0225
		Formulation X Herbicide Concentration	2.11	0.149
	2 (100)	Whole Model	13.1	**<0.0001**
		Formulation	18.5	**<0.0001**
		Glyphosate Concentration	11.2	**0.0012**
		Formulation X Glyphosate Concentration	7.04	**0.0093**
	3 (118)	Whole Model	26.1	**<0.0001**
		Glyphosate Concentration	7.73	**0.0063**
		Pelargonic Acid Concentration	14.6	**0.0002**
		Glyphosate Concentration X Pelargonic Acid Concentration	1.48	0.226

Table S5. ANOVAs for body weight of flies exposed to Roundup® starting by 2 hours after eclosion. Results of ANOVAs testing the effects of formulation, total herbicide concentration, glyphosate concentration, pelargonic acid concentration, and the interaction between these on body weight of flies exposed to herbicide within 2 hours of eclosion (DF = 127). ANOVA uses least square means identity matrix. For whole model, F-value based on Wilks' Lambda. Other F-values based on Exact F Test. Significant *p*-values after Bonferoni correction for three models ($p < 0.017$) shown in bold.

Model	Independent Variable	F	*p*
1	Whole Model	8.03	**<0.0001**
	Formulation	12.9	**0.0005**
	Herbicide Concentration	4.56	0.0349
	Formulation X Herbicide Concentration	5.66	0.0191
2	Whole Model	6.2	**0.0005**
	Formulation	0.865	0.354
	Glyphosate Concentration	3.51	0.0629
	Formulation X Glyphosate Concentration	0.933	0.336
3	Whole Model	9.13	**<0.0001**
	Glyphosate Concentration	2.8	0.0962
	Pelargonic Acid Concentration	5.63	0.0187
	Glyphosate Concentration X Pelargonic Acid Concentration	0.874	0.768

Table S6. MANOVAs for reproductive response variables of flies exposed to Roundup® starting by 2 hours after eclosion. Results of MANOVAs testing the effects of formulation, total herbicide concentration, glyphosate concentration, pelargonic acid concentration, and the interaction between these on the reproductive response variables (ovary size and number of oocytes) on flies exposed to herbicide within 2 hours of eclosion. MANOVA uses least square means identity matrix. For whole model, F-value based on Wilks' Lambda. Other F-values based on Exact F Test. Significant *p*-values after Bonferoni correction for two models (overall and by formulation; $p < 0.025$) shown in bold.

Formulation	Response Variable	*DF*	Independent Variable	F	*p*
Both Formulations	Overall	99	Whole model	8.21	**<0.0001**
			Formulation	10.1	**0.0001**
			Glyphosate Concentration	11.0	**<0.0001**
			Formulation * Glyphosate Concentration	3.49	0.034
	Ovary Volume	100	Whole model	9.43	**<0.0001**
			Formulation	19.7	**<0.0001**
			Glyphosate Concentration	1.97	**0.163**
			Formulation * Glyphosate Concentration	5.50	**0.021**
	Number of Oocytes	100	Whole model	13.1	**<0.0001**
			Formulation	18.5	**<0.0001**
			Glyphosate Concentration	11.2	**0.0012**
			Formulation * Glyphosate Concentration	7.04	**0.0093**
Super Concentrate	Overall	72	Glyphosate Concentration	1.79	0.174
	Ovary size	73	Glyphosate Concentration	0.222	0.639
	# oocytes	73	Glyphosate Concentration	0.532	0.468
Ready to Use	Overall	66	Glyphosate Concentration	18.0	**<0.0001**
	Ovary size	67	Glyphosate Concentration	27.3	**<0.0001**
	# oocytes	67	Glyphosate Concentration	35.8	**<0.0001**
